# The contribution of district prioritization on maternal and newborn health interventions coverage in rural India

**DOI:** 10.7189/jogh.10.010418

**Published:** 2020-06

**Authors:** BM Ramesh, Bidyadhar Dehury, Shajy Isac, Vikas Gothalwal, Ravi Prakash, Vasanthakumar Namasivayam, Shivalingappa Halli, James Blanchard, Ties Boerma

**Affiliations:** 1 Centre for Global Public Health, Department of Community Health Sciences, University of Manitoba, Winnipeg, Manitoba, Canada; 2 India Health Action Trust, Lucknow, Uttar Pradesh, India

## Abstract

**Background:**

In 2001, India prioritized eight most socioeconomically disadvantaged states known as Empowered Action Group (EAG) states and in 2013, it prioritized 190 of the 718 as high priority districts (HPDs) to accelerate the decline in maternal and newborn mortality. This paper assesses whether the HPDs achieved a greater coverage of maternal and newborn health interventions than the non-HPDs and HPDs in EAG states achieved greater coverage than those in non-EAG states.

**Methods:**

We used data from the Sample Registration System to assess rural neonatal mortality trends in EAG states and all India. We computed a co-coverage index based on seven maternal and newborn health interventions from the 2015/16 National Family Health Survey. Difference in differences (DID) analyses were used to examine the contribution of district prioritization, considering the HPDs and the illiterate as treatment groups and 2013 as the time cut-off for the pre- and post-treatment.

**Results:**

Neonatal mortality declined in rural India from 36 to 27 per 1000 live births during 2010-2016 at 4.5% per year. Four EAG states experienced faster rates of decline than the national rate. From 2013, the co-coverage index increased significantly more in the HPDs compared to non-HPDs (DID = 0.11, *P* ≤ 0.005). The district prioritization effect on co-coverage was statistically significant in only EAG states (DID = 0.13, *P* ≤ 0.05). The coverage gains for illiterate mothers were greater than for literate mothers, especially in the HPDs.

**Conclusions:**

The district prioritization in India is associated with greater improvements in the coverage of maternal and newborn health services in EAG states and the HPDs, including reductions in inequalities within those states and districts. There are however still large gaps between states and districts and within districts by the mother’s literacy status that need further prioritization to make progress towards the SDG targets by 2030.

Every year, globally, an estimated 303 000 mothers die during childbirth and 2.7 million babies die during the first 28 days of life [1]. India alone, with an annual estimate of 44 000 maternal and 760 000 neonatal deaths, accounts for 15 percent and 28 percent of the global maternal and neonatal deaths, respectively. Despite the recent reductions in India in both the maternal mortality ratio (from 212 maternal deaths per 100 000 live births in 2007-09 to 130 in 2014-16) [2] and the neonatal mortality rate (from 31 neonatal deaths per 1000 live births in 2011 to 24 in 2016), [3,4] the achievement of SDG targets of a maternal mortality ratio of 70 per 100 000 live births and a neonatal mortality rate of 12 per 1000 live births, by 2030 will be a challenge. In particular, this will require major efforts to improve the coverage for quality antenatal, delivery and postnatal care services in states and districts that are currently lagging behind. Among the 22 larger states in India, the neonatal mortality rate in 2016 ranged from 6 per 1000 live births in Kerala to 32 each in Madhya Pradesh and Odisha [4], and the maternal mortality ratio during 2014-16 ranged from 46 per 100 000 live births in Kerala to 237 in Assam [2].

Geographic prioritization has been one of the main strategies of the Government of India (GOI) to accelerate the decline in maternal and newborn mortalities. In 2001, GOI formed a group of eight most socioeconomically disadvantaged states, known as Empowered Action Group (EAG) states, including Bihar, Chhattisgarh, Jharkhand, Madhya Pradesh, Odisha, Rajasthan, Uttarakhand and Uttar Pradesh (**Figure 1**), accounting for about 46% of India’s population with 61% of those living below the poverty line [5,6]. For multiple health indicators, these have been identified as the poorest performing states, and national surveys generally show lower coverage for indicators of reproductive, maternal, newborn and child health in EAG states than elsewhere [7-9]. The EAG classification was used primarily for generating timely and reliable statistics at the district level through Annual Health Surveys and did not determine the resource allocation.

**Figure 1:**
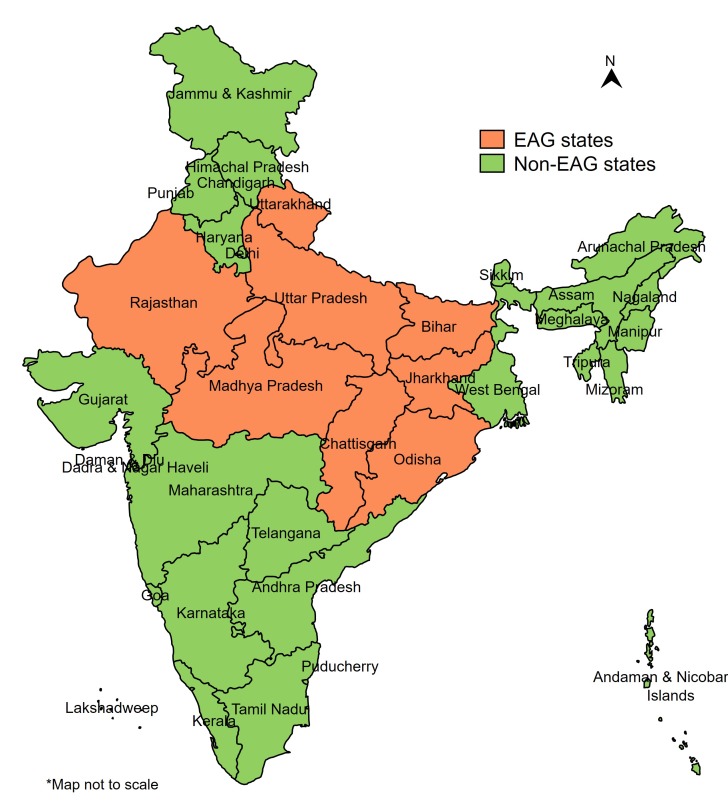
Map of India depicting Empowered Action Group (EAG) and non-EAG states.

In 2013, the GOI launched a renewed campaign to improve the reproductive, maternal, newborn, child and adolescent health (referred to as RMNCH^+^A) in 184 high priority districts (HPDs) across the country, [10] which was about one-fourth of the total 718 districts in the country. Based on a composite health index comprising maternal health, child health and family planning indicators, districts were ranked within each state and the bottom 25% of the districts as well as those affected by Left Wing Extremism were selected across 29 states. The GOI called for an integrated planning and monitoring of RMNCH^+^A interventions in these HPDs. The Government of Uttar Pradesh expanded the focus from 19 to 25 HPDs out of its 75 districts, thus increasing the total number of HPDs in the country to 190 (**Figure 2**). The strategies were developed and implemented under the National Rural Health Mission (NRHM), with a greater focus on rural populations.

**Figure 2:**
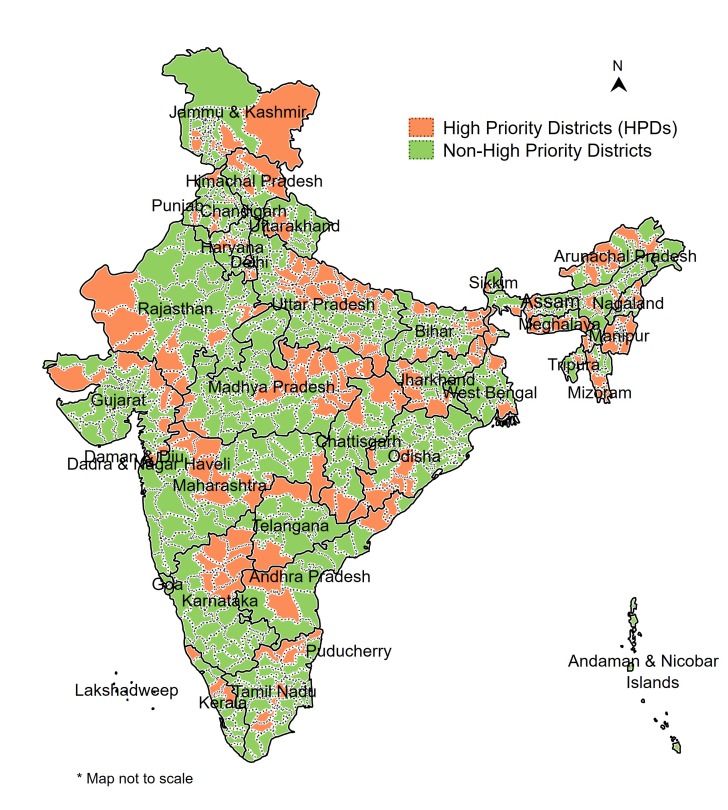
Map of India, showing high priority districts (HPDs) and non-HPDs.

The states were encouraged to allocate to the HPDs a per capita resource envelope that was at least 30% higher than in non-HPDs. The population norms were relaxed in the HPDs for ASHA (Accredited Social Health Activists, the community health workers for every 1000 population in rural areas) recruitment, and for establishment of sub-centres, upgradation of subcentres, and medical mobile units. Each state had one lead development partner assigned to serve as single point of contact and accountability. However, prioritization actions were not standardized or quantified across states, and the state actions for HPDs differed from state to state. This paper aims to assess the contribution of district prioritization on coverage of maternal and newborn health (MNH) interventions in rural India, overall and separately in EAG and non-EAG states. More specifically, it attempts to answer the following questions related to MNH coverage pre- and post-2013: (1) Have the HPDs achieved a greater coverage than the non-HPDs? (2) Have the HPDs in EAG states achieved a greater coverage than the HPDs in non-EAG states?

Area-based prioritization, which aims largely at increasing access to health services in poorly served areas in India may help reduce its high and persistent socioeconomic inequalities in health. In this paper, we also test if the HPD approach achieved a greater reduction in coverage gaps between the literate and the Illiterate. Among the many socioeconomic indicators, literacy is considered to improve both health outcomes and economic growth [11,12].

## METHODS

For setting the contexts in terms of health outcomes, we compared the rural neonatal mortality across EAG states and with India, using data from the Sample Registration System (SRS). The SRS, which is based on a system of dual recording of births and deaths in representative units spread across the country, is one of the most regular source of health statistics in India [13]. It publishes annual estimates of fertility and mortality indicators for all the states and Union Territories of India since 1971.

We used the SRS published annual rates for the neonatal mortality to assess the trends within the period 2010-2016. The annual percentage rate change (APC) during 2010-2016, and the subperiods 2010-2012 and 2013-2016 were computed to compare the speed of change in different settings. The APCs and their confidence intervals were computed using the coefficients derived from the regression of log transformed rates over years [14].

The fourth round of the National Family Health Survey (NFHS-4) [15], conducted in 2015-16, was used for coverage indicators. The NFHS is a large-scale, multi-round survey conducted in a representative sample of households throughout India. Four rounds of the survey have been completed since the first survey in 1992-93. While the first three NFHS rounds (1992-93, 1998-99, 2005-06) provided state-level estimates, the NFHS 4 (2015-16) provided district-level estimates. The annual antenatal care (ANC) and postnatal care (PNC) coverage rates were computed based on the information from NFHS-4 on the last live births during the five-year period preceding the survey. The delivery care coverage indicators were computed based on the information on the last three births in the five-year period preceding the survey. Due to the differences in the timing of the data collection in different states and districts, the calendar years for the five-year period prior to survey date corresponded to 2010 to 2016. The distribution of unweighted number of births in the five years before the survey in rural areas of EAG/non-EAG states and HPDs/non-HPDs covered in NFHS-4 is presented in Table S1 in the **Online Supplementary Document**.

We used difference in differences (DID) [16] to examine the impact of state and district prioritization in 2013 on MNH coverage. The treatment groups considered were HPDs and the illiterate. The year 2013 was used as the time cut-off for the pre- and post-treatment, as the year of initiation of the HPD focus. Selected socioeconomic variables such as caste, religion and wealth quintile were controlled for the computation of DIDs. Literacy was an additional control variable for the computation of DIDs between HPDs and non-HPDs. State fixed effects model was used for the evaluation of HPDs, so that the comparison is between HPDs and non-HPDs within each state rather than across all states. The standard errors were clustered at the district level. STATA version 14.0 was used for the analyses.

The geographies and literacy groups were compared on the trends in a co-coverage score [17], based on the total number of seven key ANC, delivery and PNC services which should be received by each individual woman. The services included: (1) any ANC (2) 3+ ANCs (3) first ANC in the first trimester (4) BP measurement during pregnancy (5) Hb measurement during pregnancy (6) institutional delivery and (7) postnatal checkup within 24 hours of delivery.

## RESULTS

### Trends in rural neonatal mortality rates across EAG states and all India

The trends in neonatal mortality for the period 2010-16 for the rural areas of EAG states and India and the APCs are shown in **Table 1**. The neonatal mortality estimates for Uttarakhand, which are available only for the period since 2014 are excluded from **Table 1**. In 2010, neonatal mortality rates were higher than India’s national rate in five of the seven EAG states, while Bihar and Jharkhand had lower neonatal mortality. During 2010-16, rural India’s neonatal mortality rate declined at an average rate of 4.5% per year. Four EAG states had significantly faster declines and one EAG state (Bihar) a significantly slower decline. The decline was fastest in Uttar Pradesh, with an APC of -5.5 (95% confidence interval (CI) = -5.6, -5.5). Jharkhand is the only EAG state that achieved a rural neonatal mortality rate lower than the national average in 2016, but its rate of decline was lower than that of UP, with an APC of -5.3 (95% CI = -5.5, -5.1). The decline in neonatal mortality rate was similar in 2013-16 compared to 2010-12. It was faster during the more recent period in only two of the EAG states – Jharkhand and Odisha.

**Table 1:** Trends in neonatal mortality rates and annual percent change (APC) in rural areas of EAG states (excluding Uttarakhand) and India, 2010-2016, SRS

Year	Bihar	Chhattisgarh	Jharkhand	Madhya Pradesh	Odisha	Rajasthan	Uttar Pradesh	India
2010	32	38	32	47	43	45	45	36
2011	31	34	31	44	42	41	43	34
2012	29	32	30	42	41	39	40	33
2013	29	31	28	39	39	36	38	31
2014	29	29	27	39	38	37	36	30
2015	29	28	25	37	36	34	34	29
2016	28	27	23	35	33	31	32	27
APC (2010-16)	-1.9 (-2.1, -1.7)	-5.3 (-5.4, -5.1)	-5.3 (-5.5, -5.1)	-4.6 (-4.7, -4.4)	-4.1 (-4.3, -4.0)	-5.4 (-5.6, -5.2)	-5.5 (-5.6, -5.5)	-4.5 (4.5, -4.4)
APC (2010-12)	-3.6 (-3.8, -3.3)	-6.5 (-6.9, -6.1)	-4.2 (-4.4, -4.0)	-5.9 (-6.0, -5.8)	-3.1 (-3.3, -3.0)	-6.9 (-7.1, -6.8)	-5.6 (-5.7, -5.5)	-4.7 (-4.8, -4.5)
APC (2013-16)	-1.0 (-1.2, -0.9)	-4.4 (-4.6, -4.2)	-6.5 (-6.7, 6.2)	-3.7 (-4.0, -3.4)	-5.4 (-5.7, -5.1)	-5.2 (-5.8, -4.6)	-5.6 (-5.6, -5.5)	-4.4 (-4.6, -4.2)

SRS – Sample Registration System

### Contribution of HPD approach on coverage of key MNH services

The differences in differences (DID) in co-coverage index for the rural populations between HPDs and non-HPDs in EAG and non-EAG states are presented in **Table 2**. The district prioritization had a positive effect on the co-coverage index. During 2013-16, the mean number of key MNH services received increased significantly more in EAG than in non-EAG states (DID = 0.32, *P* ≤ 0.005). The impact of district prioritization on the co-coverage index was smaller, with a DID for HPDs/Non-HPDs of 0.08 (*P* ≤ 0.005). The effect of HPD/non-HPD approach on co-coverage index was statistically significant in only the EAG states (DID = 0.10, *P* ≤ 0.005) compared to the non-EAG states (DID = -0.04, *P* > 0.05). Among the births during 2013-2016 in NFHS-4, the mean number of MNH services received ranged from 3.60 (95% CI = 3.33, 3.87) in HPDs of EAG states to 5.97 (95% CI = 5.88, 6.05) in non-HPDs of non-EAG states.

**Table 2:** Co-coverage score of key ANC, delivery and PNC services received among the last births in the five years preceding the survey and the differences in differences (DID) between the EAG and non-EAG states and between the HPDs and Non-HPDs, NFHS-4

Comparison groups	Birth year: 2010-2012		Birth year: 2013-2016		DID (95% CI)
**Co-coverage score (95% CI)**	**Number of cases**	**Co-coverage score (95% CI)**	**Number of cases**
EAG states	3.60 (3.42, 3.78)	23708	4.10 (3.93, 4.27)	58304	0.32† (0.27, 0.38)
Non-EAG states	5.63 (5.52, 5.75)	19535	5.75 (5.64, 5.86)	41436	
HPDs	3.99 (3.67, 4.31)	14193	4.25 (4.00, 4.50)	34664	0.08† (0.03, 0.13)
Non-HPDs	4.87 (4.69, 5.06)	29050	5.03 (4.86, 5.19)	65076	
HPDs in EAG states	3.02 (2.76, 3.28)	8090	3.60 (3.33, 3.87)	20086	0.10† (0.03, 0.16)
Non-HPDs in EAG states	3.91 (3.70, 4.13)	15618	4.37 (4.17, 4.57)	38218	
HPDs in non-EAG states	5.20 (4.94, 5.45)	6103	5.30 (5.05, 5.54)	14578	0.04 (-0.02, 0.11)
Non-HPDs in non-EAG states	5.81 (5.72, 5.91)	13432	5.97 (5.88, 6.05)	26858	

ANC – antenatal care, PNC – post-natal care, NFHS-4 – National Family Health Survey 4, CI – confidence interval, DID – difference in differences, EAG – Empowered Action Group, HPD – high priority district

*The standard errors for the means are clustered at the district level. The DIDs are computed controlling for selected socioeconomic variables including caste, religion, wealth index and literacy. State fixed effects are considered while computing the DIDs for HPDs and non-HPDs. Figures in parentheses denote 95% confidence intervals.

†*P* ≤ 0.005.

The HPD focus was not associated with closing the coverage gap between the literates and the illiterates in India, particularly among births in 2013 and later (**Table 3**). Overall, the increase in the average number of key MNH services received was greater among the illiterate mothers than the literate mothers (DID = 0.22, *P* ≤ 0.005) beginning 2013. Although the gain among the illiterate was greater than among the literate in all geographies, the DID was the same in HPDs and non-HPDs, irrespective of the EAG status (0.14, *P* ≤ 0.005). The coverage gaps between the literate and the illiterate was reduced more in EAG states (DID = 0.15, *P* ≤ 0.005) than in non-EAG states (DID = 0.03, *P* > 0.005). However, the DIDs comparing the literate and the illiterate in HPDs and non-HPDs in EAG and non-EAG states were not significant, except for the non-HPDs in EAG states. Among births in 2013-2016, the mean number of key MNH services received ranged from 3.06 (95% CI = 2.80, 3.33) for the illiterate in HPDs in EAG states to 6.11 (95% CI = 6.03, 6.18) for the literates in non-HPDs in non-EAG states.

**Table 3:** Co-coverage score of key ANC, delivery and PNC services received among the last births in the five years preceding the survey and the differences in differences between the illiterate and the literate according to the state and district groupings, NFHS-4

Comparison groups	Birth year: 2010-2012		Birth year: 2013-2016		DID (95% CI)
**Co-coverage score (95% CI)**	**Number of cases**	**Co-coverage score (95% CI)**	**Number of cases**
Illiterate	3.47 (3.29, 3.67)	17042	3.77 (3.61, 3.94)	36934	0.22† (0.15, 0.29)
Literate	5.29 (5.17, 5.41)	26201	5.35 (5.25, 5.46)	62806	
Illiterate in EAG states	2.89 (2.72, 3.06)	12107	3.41 (3.23, 3.58)	26796	0.15† (0.07, 0.23)
Literate in EAG states	4.39 (4.22, 4.56)	11601	4.73 (4.58, 4.87)	31508	
Illiterate in non-EAG states	4.84 (4.63, 5.04)	4935	4.95 (4.75, 5.15)	10138	0.03 (-0.09, 0.14)
Literate in non-EAG states	5.88 (5.79, 5.98)	14600	5.98 (5.89, 6.06)	31298	
Illiterate in HPDs	3.03 (2.74, 3.32)	6987	3.41 (3.15, 3.66)	15680	0.14† (0.06, 0.22)
Literate in HPDs	4.90 (4.65, 5.16)	7206	4.98 (4.77, 5.19)	18984	
Illiterate in non-HPDs	3.78 (3.55, 4.01)	10055	4.04 (3.83, 4.24)	21254	0.14† (0.08, 0.19)
Literate in non-HPDs	5.43 (5.30, 5.56)	18995	5.50 (5.38, 5.62)	43822	
Illiterates in HPDs in EAG states	2.49 (2.25, 2.73)	4968	3.06 (2.80, 3.33)	11109	0.11 (-0.01, 0.22)
Literates in HPDs in EAG states	3.89 (3.59, 4.19)	3122	4.31 (4.05, 4.57)	8977	
Illiterates in HPDs in non-EAG states	4.34 (3.94, 4.74)	2019	4.46 (4.09, 4.82)	4571	0.07 (-0.05, 0.20)
Literates in HPDs in non-EAG states	5.60 (5.39, 5.81)	4084	5.65 (5.44, 5.86)	10007	
Illiterates in non-HPDs in EAG states	3.17 (2.95, 3.39)	7139	3.65 (3.43, 3.87)	15687	0.14† (0.07, 0.22)
Literates in non-HPDs in EAG states	4.58 (4.38, 4.77)	8479	4.90 (4.73, 5.07)	22531	
Illiterates in non-HPDs in non-EAG states	5.16 (4.98, 5.33)	2916	5.33 (5.18, 5.48)	5567	0.03 (-0.06, 0.11)
Literates in non-HPDs in non-EAG states	5.98 (5.89, 6.08)	10516	6.11 (6.03, 6.18)	21291	

ANC – antenatal care, PNC – post-natal care, NFHS-4 – National Family Health Survey 4, CI – confidence interval, DID – difference in differences??, EAG – Empowered Action Group, HPD – high priority district

*The standard errors for the means are clustered at the district level. The DIDs are computed controlling for selected socioeconomic variables including caste, religion and wealth index. State fixed effects are considered while computing the DIDs for HPDs and non-HPDs. Figures in parentheses denote 95% confidence intervals.

†*P* ≤ 0.005.

**Figure 3:**
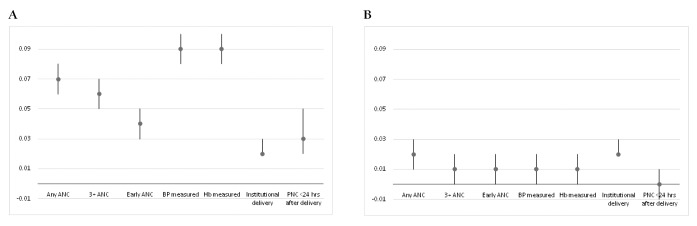
Difference in differences (DID) in the coverage of specific antenatal care (ANC), delivery and postnatal care (PNC) services. **Panel A.** Difference in differences (DID) between EAG and non-EAG states in the coverage of specific ANC, delivery and PNC services, among rural births before 2013 and from 2013-16, National Family Health Survey 4 (NFHS-4). Note: DIDs are adjusted for caste, religion, wealth quintile and literacy. **Panel B.** Difference in differences (DID) between HPDs and non-HPDs in the coverage of specific ANC, delivery and PNC services, among rural births before 2013 and from 2013-16, NFHS-4. Note: DIDs are adjusted for caste, religion, wealth quintile and literacy, considering the state fixed effects.

**Figure 4:**
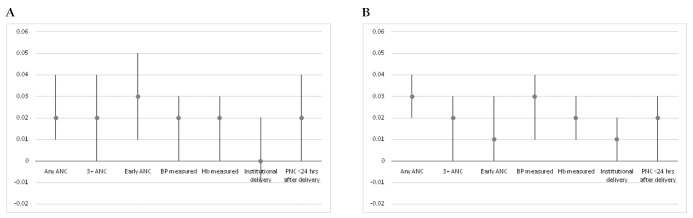
Difference in differences (DID) between the literate and the illiterate in the coverage of specific antenatal care (ANC), delivery and postnatal (PNC) services, among rural births. **Panel A.** Difference in differences (DID) between the literate and the illiterate in the coverage of specific ANC, delivery and PNC services, among rural births in HPDs before 2013 and from 2013-16, National Family Health Survey 4 (NFHS-4). Note: DIDs are adjusted for caste, religion, and wealth quintile, considering the state fixed effects. **Panel B.** Difference in differences (DID) between the literate and the illiterate in the coverage of specific ANC, delivery and PNC services, among rural births in non-HPDs before 2013 and from 2013-16, NFHS-4. Note: DIDs are adjusted for caste, religion, and wealth quintile, considering the state fixed effects.

Of the seven key MNH services considered in this paper, the relative gains for the EAG states from 2013 were larger for four services: any ANC, 3+ ANC visits, blood pressure and Hb measurements (**Figure 3**, Panel A). For each of the key services, the relative gains for the HPDs were lower than those for the EAG states and were similar across the continuum of care (**Figure 3**, Panel B). However, the relative gains in the coverage for each key service for the illiterate over the literate from 2013-2016 were somewhat similar in HPDs and non-HPDs (**Figure 4**, Panel A and Panel B).

## DISCUSSION

Rural India has experienced a major decline in neonatal mortality from 36 to 27 per 1000 live births during 2010-2016 at 4.5% per year. However, at this rate of decline India would reach the 2030 SDG target of 12 per 1000 live births only by 2034. Four of the seven EAG states experienced a faster rate of decline than the national rate, reducing the gap with other states. There was no acceleration of the neonatal mortality decline in the EAG states from 2013 even though intervention coverage increased significantly.

Equal access to health care and equal health outcomes are an essential goal of universal health coverage strategies. While equal access relates to horizontal equity where equals are given equal treatment, equal health outcomes relates to vertical equity where those who are different in health outcomes are treated proportionately differently [18]. Prioritization of certain groups in society on the basis of their background, including where they live, is one the strategies adopted [19,20]. India’s strategy to prioritize the slow-progressing states and districts seems to have dual purposes: to accelerate country’s progress towards global goals and targets as well as to achieve health equity. Since the health care systems in India are organized by states, districts and blocks, the issues of geographic equity become important in GOI’s policies related to the distribution of resources for health.

Scientific literature on the evaluation of geographic prioritization based on health outcomes has been limited. Even within the Indian context, most published studies are on coverage of health services and health outcomes in EAG states themselves [21-24]; and comparisons of EAG and non-EAG states on the progress made on coverage of critical MNH services have been limited. Our analyses show that there have been greater improvements in EAG states in the coverage for key MNH services compared to the non-EAG states between the pre-2013 period and the period from 2013. Although the EAG classification of states was done almost two decades ago, no differential resource allocations were made on the basis of EAG status. The purpose of this classification was primarily to have a more intensive monitoring, through additional data collection, in the EAG states. For example, the three rounds of Annual Health Surveys were carried out only in EAG states plus Assam. However, in 2013, when the HPDs were identified, greater resources and administrative flexibilities were accorded to the HPDs, along with a more intensified and coordinated support from development partners.

The district prioritization has also contributed to a greater improvement in HPDs than in non-HPDs in the coverage for key MNH services between the two periods – before and after the identification of high priority districts. However, the contribution of district prioritization has been greater in EAG states than the non-EAG states. The overall contribution of geographic prioritization was greater at the state level (DID between EAG and non-EAG states) than at the district level (DID between HPDs and non-HPDs), suggesting that the greater progress in EAG states was only partially due to the faster improvements in their HPDs.

The contribution of state prioritization has been greater on components of ANC (except early ANC) than on other services in the maternal and newborn care continuum. However, the contribution of district prioritization has been almost the same for the different components of MNH services.

Geographic prioritization in India has also contributed to greater improvements in the marginalized groups. There have been greater gains for the illiterate mothers than the literates in the coverage of key MNH services. The gains for the illiterate have been substantially greater in HPDs than the non-HPDs. There are however still large differences by literacy and much remains to be done to reach equitable levels of coverage of essential interventions for all women.

There are several limitations to the study. First, no data on actual health expenditure and program implementation in the states and districts were available, limiting the actual data on exposure to the “treatment”. The time lag between the policy decision to prioritize districts and the actual implementation of the policy varied from state to state. We used 2013 as the cut off point for district prioritization at the policy level, and the focus on high priority districts continued even into the 2019-20 project implementation plans with large inter-state differences in the implementation of the policy. Second, districts with lower base level of health outcomes and service coverage are likely to have a different growth trajectory than those with higher base levels, and it is a challenge to test the DID assumption of whether the two groups of treatment and controls would have followed the same growth trajectory had the treatment not taken place. Third, our analysis does not prove causality, but our conclusions are based on the observations that, by and large, EAG states and high priority districts in EAG states have made greater progress than other states and districts. Fourth, our analyses of coverage trends are derived from retrospective data collected in a single survey. Therefore, the data referring to the period 2010-12 are based on a longer recall period and from older women than for 2013-16, with potential biases. Another limitation of this study is that due to the substantial difference in the timing of data collection for NFHS-4 across states and districts, the births at each end of the calendar year range in each state do not represent all districts, thus reducing the representativeness of the samples in those calendar years.

## CONCLUSIONS

The state and district prioritization for RMNCH^+^A in India has contributed to greater improvements in the coverage of MNH services in poor performing geographies such as the EAG states and the HPDs. Greater resources and implementation support to prioritized geographies is also likely to reduce the coverage gaps in the most marginalized groups, thus resulting in greater equity. There are however still large gaps between states and districts and within districts by the mother’s literacy status that need further prioritization in the coming years to be able to reach the SDG targets by 2030 including equity.

## Additional material

Online Supplementary Document
