# Supplementary material for: The contribution of district prioritization on maternal and newborn health interventions coverage in rural India
Source: J Glob Health. 2020 Apr 15;10(1):010418. doi: 10.7189/jogh.10.010418 (PMC7182352; doi:10.7189/jogh.10.010418)
Supplement: Online Supplementary Document [file jogh-10-010418-s001.pdf]

Table S1: Distribution of unweighted number of births in rural areas in the five years preceding the survey by year of birth, according to EAG and HPD status, NFHS-4, 2015-16.

Birth year	EAG states	Non-EAG states	HPDs	Non-HPDs	Total
2010	7762	4373	4500	7635	12135
2011	18976	12980	11142	20814	31956
2012	23987	16786	14410	26363	40773
2013	23317	16415	13872	25860	39732
2014	23973	16499	14174	26298	40472
2015	15509	11071	9223	17357	26580
2016	4359	2241	2517	4083	6600
Total	117883	80365	69838	128410	198248
